# Neuroanatomical and Psychosocial Effects of Heavy Alcohol Use Among Young Adults in Angola: A Cross‐Sectional Study

**DOI:** 10.1002/hsr2.71358

**Published:** 2025-10-06

**Authors:** Bevaldo de Oliveira Guerra, Celestino Delgado, Euclides Nenga Manuel Sacomboio

**Affiliations:** ^1^ Faculty of Medicine of the Jean Piaget University of Angola Luanda‐Angola; ^2^ Institute of Health Sciences of the Agostinho Neto University (ISCISA/UAN) Luanda‐Angola; ^3^ Higher Institute of Health Sciences of the Catholic University of Angola (ISCS/UCAN) Luanda‐Angola

**Keywords:** Angola, cortical atrophy, heavy alcohol use, neurological effects, social stigma, young adults

## Abstract

**Background and Aims:**

Excessive alcohol consumption is a critical yet under‐researched public health problem in Angola, particularly among young adults. Although per capita consumption is among the highest in Africa, little is known about the specific neuroanatomical and psychosocial consequences in this group. This study aimed to evaluate sociodemographic, neuroanatomical, and psychosocial factors associated with heavy alcohol consumption among young adults in the Rangel district, Luanda, with emphasis on cranial tomography findings and psychosocial outcomes.

**Methods:**

We conducted an analytical cross‐sectional study with a non‐probabilistic convenience sample of 61 young adults aged 18–35 years. Data were collected through structured questionnaires and cranial CT scans at Prenda Hospital. Associations were tested using chi‐square (two‐sided, *α* = 0.05).

**Results:**

Heavy alcohol consumption was identified in 59.0% (95% CI: 46.3–71.0) of participants, predominantly among males (77.8%; 95% CI: 60.9–89.9), those aged 30–35 years (83.3%), and those with education at or below high school level (96.0%). CT scans showed a high prevalence of general cortical atrophy (71.4%; *p* < 0.001) and significant parietal lobe atrophy (100%; *p* = 0.043) among heavy drinkers. Temporal and frontal lobe atrophy were observed but were not statistically significant. Neurological symptoms included memory loss, tremors, and aggressiveness, while social consequences included stigma (*p* < 0.001) and unemployment.

**Conclusion and Recommendations:**

Heavy alcohol use among young adults in Rangel is linked to sociodemographic vulnerability and significant neuroanatomical damage, particularly in the parietal lobe. Public health strategies should combine school‐based prevention, early screening, neurocognitive rehabilitation, and stigma reduction, alongside strict enforcement of alcohol sales regulations.

## 1 Introduction

Alcohol misuse is a major global public health issue, responsible for 5.3% of all deaths and 5.1% of the global burden of disease. In sub‐Saharan Africa, and particularly in Angola, alcohol consumption rates are among the highest, with the WHO estimating a per capita consumption of 10.4 liters of pure alcohol per year. Young adults are disproportionately affected due to early initiation, peer influence, and lack of preventive policies. International agencies such as the WHO and the African Union have promoted strategies including taxation, advertising restrictions, and community education, but in Angola, implementation remains limited and enforcement weak. Government campaigns exist but are sporadic, underfunded, and not youth‐focused, leaving significant gaps in prevention and treatment [1, 2].

In Angola, alcohol consumption is both culturally embedded and socially tolerated, particularly among men and young adults. Reports from the World Health Organization indicate that Angola is among the African countries with the highest per capita alcohol consumption, with limited regulation and low public awareness about the associated harms [3, 4, 5]. Local studies have highlighted a pattern of early initiation, binge drinking, and high usage of locally brewed beverages with high alcohol content [5].

These trends are especially concerning in socioeconomically vulnerable areas like the Rangel district of Luanda, where youth face high unemployment rates, limited access to education, and scarce mental health services. Social norms, peer pressure, and lack of preventive policies further reinforce harmful alcohol use. Despite the scale of the problem, little is known about the neurological or psychosocial consequences of excessive alcohol consumption in this context.

The neurotoxic effects of chronic alcohol use are well established. Alcohol disrupts the central nervous system through direct cellular toxicity, thiamine deficiency, and oxidative stress, resulting in cortical atrophy, white matter degradation, and structural damage in key areas such as the frontal, temporal, and parietal lobes [6, 7, 8]. These changes contribute to deficits in memory, learning, motor control, and social behavior. Neuroimaging studies have consistently demonstrated alcohol‐related brain alterations in long‐term users, particularly in the form of reduced cortical volume and subcortical lesions [9, 10, 11].

While global guidelines such as those from the World Health Organization define heavy drinking as consumption exceeding 60 g of pure alcohol per day for men (and 40 g for women), these definitions are rarely applied in clinical or public health settings in Angola [12, 13].

Given this gap in regional data, the present study aims to evaluate the sociodemographic factors associated with excessive alcohol consumption among young people living in the Rangel district of Luanda, Angola. In particular, the study investigates cranial computed tomography (CT) findings and psychosocial impacts to provide a comprehensive understanding of alcohol‐related harm in this under‐researched population.

## 2 Methodology

### 2.1 Study Design and Setting

This was a cross‐sectional analytical study with a quantitative approach, conducted between March and August 2022 in the Rangel district of Luanda, Angola. The study aimed to evaluate sociodemographic, neuroanatomical, and psychosocial factors associated with heavy alcohol consumption among young adults in Rangel district, with emphasis on cranial tomography findings and psychosocial consequences.

### 2.2 Sample and Participant Selection

A total of 61 participants aged 18–35 years were selected using a non‐probabilistic, convenience sampling method. Although the sample size was limited, it was deemed sufficient for exploratory analysis of preliminary associations in an under‐researched population. This limitation is acknowledged in the discussion.

*Inclusion criteria were:* (a) age between 18 and 35 years, (b) self‐reported regular consumption of alcoholic beverages, (c) residence in the Rangel district, (d) agreement to undergo cranial computed tomography (CT), and signed informed consent.

*Exclusion criteria included:* (a) pregnancy (first trimester), (b) history of prior cranial surgery or trauma, (c) contraindications to CT (e.g., metallic implants), (d) refusal or logistical impossibility to undergo the CT scan.

Participants were selected using a non‐probabilistic, convenience sampling method due to the absence of a complete sampling frame and the need for feasibility in a resource‐limited setting. This method, while limiting generalizability, allowed the timely recruitment of eligible participants within the study period. Participants who completed the survey but did not undergo CT were excluded from imaging analyses due to the limited availability of scanning appointments and occasional participant refusal.

### 2.3 Definition of Heavy Alcohol Consumption

We defined “heavy drinking” based on WHO guidelines: an average consumption exceeding 60 grams of pure ethanol per day for men and 40 grams per day for women [13].

### 2.4 Data Collection Instruments

Data were collected using a structured questionnaire (“data collection grid”) developed by the research team, with validation by clinical experts. It included items covering:

*Sociodemographic profile (age, sex, education level, employment)*,

Alcohol consumption (type, frequency, duration, quantity),

Neurological symptoms (e.g., tremors, memory loss, aggression),

Family and social impacts (e.g., abandonment, unemployment, stigma).

Socioeconomic status was assessed using indicators of income, occupation, and education. Psychosocial impacts were measured using direct questions about the personal and social consequences of drinking. The questionnaire did not employ validated scales due to limitations in contextual adaptation.

### 2.5 Cranial Tomography Procedure and Image Analysis

Cranial CT scans were performed at Prenda Hospital using a Siemens SOMATOM Emotion 16‐slice scanner. Image interpretation was conducted using RadiAnt DICOM Viewer software (version 2024.1).

All images were analyzed by a board‐certified radiologist with over 10 years of experience in neuroradiology. We acknowledge that using a single evaluator introduces a potential source of bias, which is included in the limitations section.

### 2.6 Statistical Analysis

Data were entered into Microsoft Excel and analyzed using IBM SPSS Statistics (version 20). Descriptive statistics were used to summarize the data. Associations between categorical variables were tested using the chi‐square test. Categories with low expected counts were merged where appropriate to meet the assumptions of the test. Corrections for multiple comparisons were applied using the Bonferroni method.

All tests were two‐sided, and statistical significance was set at *α* = 0.05. *P*‐values were reported by current guidelines:

*p* < 0.001 for highly significant results.

Three decimal places for 0.001 ≤ *p* < 0.01.

Two decimal places for *p* ≥ 0.01.

*p* > 0.99 when applicable.

Effect sizes (e.g., risk difference or odds ratios) were reported when relevant. No imputation was performed for missing data.

### 2.7 Ethical Considerations

The study was approved by the Ethics Committee of Jean Piaget University of Angola (Ref. No. 111/CC/FM/UNPIAGET/2022) and authorized by Prenda Hospital (Ref. No. 172/DPC/HP/2022). All participants signed informed consent forms before enrollment. The study adhered to the principles of the Declaration of Helsinki. Confidentiality and anonymity were preserved throughout the study.

### 2.8 Limitations

This study has several limitations. The small sample size (*n* = 61) and non‐probabilistic sampling reduce the generalizability of findings. A power analysis was not performed; future studies should aim to determine minimum sample sizes to ensure adequate statistical power. Cross‐sectional tomographic data prevent causal inferences. Self‐reported data may be affected by recall bias or underreporting. The use of CT, while accessible, may be less sensitive than MRI in detecting subtle neural changes.

## 3 Results

Among the 61 participants included in the study, over half (59.0%) were classified as heavy drinkers according to WHO criteria. This pattern was more frequent among males, older young adults, and those with lower educational attainment. The following sections summarize these associations, beginning with sociodemographic characteristics (Table 1).

**Table 1:** Relationship between heavy consumption of alcoholic beverages versus Sociodemographic data.

Sociodemographic data	Heavy drinkers (%)	Nonheavy *n* (%)	Total *n* (%)	*χ*² (*df*)	*p* value
Overall	36 (59.0)	25 (41.0)	61 (100)	—	—
Sex					
Male	28 (77.8)	8 (22.2)	36 (59.1)	12.67 (1)	***p*** < 0**.001**
Female	8 (32.0)	17 (68.0)	25 (40.9)	—	—
Age Range					
18–23 y	9 (39.1)	14 (60.9)	23 (37.7)	11.00 (2)	***p*** = **0.004**
24–29 y	17 (65.4)	9 (34.6)	26 (42.6)	—	—
30–35 y	10 (83.3)	2 (16.7)	12 (19.7)	—	—
Education level					
≤ High‐school*	24 (96.0)	1 (4.0)	25 (41.0)	24.56 (1)	***p*** < **0.001**
Higher education	12 (33.3)	24 (66.7)	36 (59.0)	—	—
Type of alcoholic drink					
Beer	41 (89.1)	5 (10.9)	46 (75.4)	9.59 (1)	***p*** = **0.002**
Wine	29 (82.9)	6 (17.1)	35 (57.3)	0.33 (1)	*p* = 0.564
Spirits	32 (91.4)	3 (8.6)	35 (57.3)	6.39 (1)	***p*** = 0**.011**
Mixed drinks	40 (95.2)	2 (4.8)	42 (68.9)	14.67 (1)	***p*** < 0**.001**

*Note:* Bold values are indicate *p* < 0.05.

A significant association was observed between sex and alcohol consumption patterns. Heavy drinking was more prevalent among males (77.8%) compared to females (32.0%) (*χ*²(1) = 12.67; *p* < 0.001). This indicates a strong gender disparity, with men being significantly more likely to consume alcohol excessively.

The age group also demonstrated a statistically significant association with heavy drinking (*χ*²(2) = 11.00; *p* = 0.004). The prevalence of heavy consumption increased with age: 39.1% in the 18–23 age group, 65.4% in the 24–29 age group, and 83.3% among those aged 30–35. This trend suggests a cumulative exposure or greater social autonomy among older young adults.

Education level showed a highly significant inverse association with heavy alcohol use. While 96.0% of participants with education at or below high school level were heavy drinkers, only 33.3% of those with higher education reported excessive consumption (*χ*²(1) = 24.56; *p* < 0.001). This finding suggests that higher educational attainment may serve as a protective factor against harmful alcohol use.

Regarding the type of alcoholic beverage, significant associations were found for beer (*χ*²(1) = 9.59; *p* = 0.002), spirits (*χ*²(1) = 6.39; *p* = 0.011), and mixed alcoholic drinks (*χ*²(1) = 14.67; *p* < 0.001), with a large proportion of heavy drinkers among their consumers. Conversely, wine consumption did not show a significant association (*χ*²(1) = 0.33; *p* = 0.564), possibly reflecting lower intake volumes or differing patterns of use.

Table 2 shows that among the 49 participants who underwent cranial computed tomography (CT), 35 (71.4%) of the heavy drinkers exhibited general cortical atrophy. The association between heavy alcohol use and overall brain atrophy was statistically significant (*χ*²(1) = 13.36; *p* < 0.001).

**Table 2:** Relationship between heavy drinking and tomographic findings.

Data on tomographic findings	Yes	No	Total	Chi‐Square (*χ*²)	*p* value
All (CT sample)	36 (73.5%)	13 (26.5%)	49 (100%)	—	—
Brain Atrophies					
General atrophy	35 (71.4%)	14 (28.6%)	49 (100%)	13.36	***p*** < **0.001**
Parietal atrophy	12 (100%)	0 (0.0%)	12 (24.5%)	4.08	***p*** = **0.043**
Temporal atrophy	29 (78.4%)	8 (21.6%)	37 (75.5%)	0.98	*p* = 0.322
Frontal atrophy	33 (70.2%)	14 (29.8%)	47 (95.9%)	0.20	*p* = 0.652
Cerebellar atrophy	3 (100%)	0 (0.0%)	3 (6.1%)	0.16	*p* = 0.690
Other Tomographic findings					
Thickening of the turbinate mucosa	7 (87.5%)	1 (12.5%)	8 (16.3%)	0.30	*p* = 0.586
Destruction of the middle turbinates	2 (100%)	0 (0.0%)	2 (4.1%)	0.00	*p* = 0.960
Focal lesion in the basal ganglia	1 (100%)	0 (0.0%)	1 (2.0%)	0.00	*p* = 1.000
Lacunar infarction (basal ganglia ‐ CVA)	0 (0.0%)	1 (100%)	1 (2.0%)	0.29	*p* = 0.591

*Note:* Bold values are indicate *p* < 0.05.

More specifically, alcohol consumption was significantly associated with atrophy in certain cerebral regions. Parietal lobe atrophy was found in 100% of affected individuals, with a significant association (*χ*²(1) = 4.08; *p* = 0.043). Temporal lobe atrophy was present in 78.4% of cases, though the association was not statistically significant (*χ*²(1) = 0.98; *p* = 0.322). Similarly, frontal lobe atrophy was observed in 70.2% of heavy drinkers but without a significant association (*χ*²(1) = 0.20; *p* = 0.652).

Cerebellar atrophy was identified in only three participants (100%), all of whom were heavy drinkers; however, the small sample size likely limited the statistical power of the test (*χ*²(1) = 0.16; *p* = 0.690). Regarding other tomographic findings, thickening of the turbinate mucosa was observed in 87.5% of cases (*χ*²(1) = 0.30; *p* = 0.586), and destruction of the middle turbinates occurred in two cases (*p* = 0.960), with no significant associations. One focal lesion in the basal ganglia and one lacunar infarction (CVA) were documented, neither of which showed statistically significant relationships with heavy alcohol use (*p* = 0.401 and *p* = 0.591, respectively).

These results highlight a predominant pattern of alcohol‐related cortical atrophy, particularly in the parietal lobe, while underscoring the limitations of sample size in detecting other structural abnormalities.

Table 3 shows, heavy alcohol consumption was significantly associated with a variety of self‐reported neurological symptoms.

**Table 3:** Relationship between cortical atrophy and neurological, family, and social consequences.

Conseqnences	Yes	No	Total	Chi‐Square (*χ*², *df*)	*p* value
All participants	36 (59.0%)	25 (41.0%)	61 (100%)	—	—
Neurological					
Learning deficit	16 (100%)	0 (0.0%)	16 (26.2%)	12.85 (1)	***p*** < **0.001**
Memory loss	16 (100%)	0 (0.0%)	16 (26.2%)	12.85 (1)	***p*** < **0.001**
Decreased visual acuity	11 (100%)	0 (0.0%)	11 (18.1%)	7.37 (1)	***p*** = **0.007**
Excessive sleepiness	12 (100%)	0 (0.0%)	12 (19.6%)	8.37 (1)	***p*** = **0.004**
Loss of balance	9 (90.0%)	1 (10.0%)	10 (16.3%)	3.34 (1)	*p* = 0.068
Insomnia	5 (100%)	0 (0.0%)	5 (8.1%)	2.16 (1)	*p* = 0.142
Aggressiveness	19 (100%)	0 (0.0%)	19 (31.1%)	16.78 (1)	***p*** < **0.001**
Tremors	15 (100%)	0 (0.0%)	15 (24.5%)	11.66 (1)	***p*** < **0.001**
Family					
Abandonment	4 (100%)	0 (0.0%)	4 (6.5%)	1.44 (1)	*p* = 0.231
Disruption	1 (100%)	0 (0.0%)	1 (1.6%)	0.00 (1)	*p* = 1.000
Isolation	15 (100%)	0 (0.0%)	15 (24.5%)	11.66 (1)	***p*** < **0.001**
No family consequences	29 (70.7%)	12 (29.3%)	41 (67.2%)	5.70 (1)	***p*** = **0.017**
Social					
Unemployment	5 (100%)	0 (0.0%)	5 (8.1%)	2.16 (1)	*p* = 0.142
Rejection	2 (100%)	0 (0.0%)	2 (3.2%)	0.22 (1)	*p* = 0.640
Stigma	16 (100%)	0 (0.0%)	16 (26.2%)	12.85 (1)	***p*** < **0.001**
Any consequences (yes)	26 (68.4%)	12 (31.6%)	38 (62.2%)	2.73 (1)	*p* = 0.099

*Note:* Bold values are indicate *p* < 0.05.

All participants who reported learning deficits (*n* = 16) and memory loss (*n* = 16) were classified as heavy drinkers. These associations were statistically significant (*χ*²(1) = 12.85; *p* < 0.001 for both symptoms). Similarly, tremors (*χ*²(1) = 11.66; *p* < 0.001) and aggressiveness (*χ*²(1) = 16.78; *p* < 0.001) were exclusively reported among heavy drinkers.

Other symptoms, including excessive sleepiness (*n* = 12; *χ*²(1) = 8.37; *p* = 0.004) and decreased visual acuity (*n* = 11; *χ*²(1) = 7.37; *p* = 0.007), also showed significant associations. Loss of balance (*n* = 10; *χ*²(1) = 3.34; *p* = 0.068) approached statistical significance, while insomnia (*n* = 5; *p* = 0.142) did not show a meaningful association.

Regarding family consequences, social isolation (*χ*²(1) = 11.66; *p* < 0.001) was significantly associated with heavy alcohol use. Conversely, abandonment (*p* = 0.231) and family disruption (*P* = 1.000) were not statistically significant, likely due to low event frequency. Notably, participants who reported no family consequences were significantly less likely to be heavy drinkers (*χ*²(1) = 5.70; *p* = 0.017).

In the social domain, the experience of stigma showed a strong association with heavy alcohol use (*χ*²(1) = 12.85; *p* < 0.001). Although unemployment (*n* = 5) was reported exclusively among heavy drinkers, the association did not reach statistical significance (*p* = 0.142), likely due to the small sample size. Rejection and broader social exclusion were also reported, but without significant associations.

## 4 Discussion

The findings of this study underscore the multifactorial and structural determinants of heavy alcohol consumption among young adults in Angola and their significant neuroanatomical and psychosocial consequences. The predominance of alcohol misuse among men aged 24–35 with lower levels of education mirrors global and regional trends linking harmful alcohol use to poverty, unemployment, low educational attainment, and chronic exposure to psychosocial stressors such as violence and marginalization [12, 13, 14].

From a neurodevelopmental perspective, emerging adulthood represents a period of heightened vulnerability to substance use. The prefrontal cortex, critical for executive functions including impulse control, risk evaluation, and decision‐making, undergoes significant maturation during this stage [15]. Alcohol disrupts these neurobiological processes, impairing judgment and increasing the likelihood of risk‐taking behaviors. The high prevalence of frontal lobe atrophy observed in our sample supports this vulnerability, consistent with neuroimaging studies demonstrating alcohol‐induced disruption of synaptic pruning and myelination in prefrontal regions [16, 17].

Parietal lobe atrophy was statistically significant and aligns with literature linking this region to impairments in sensory integration, spatial orientation, and motor coordination [18]. Temporal lobe atrophy, although frequently observed, did not reach statistical significance in our sample and should be interpreted cautiously [19]. These structural changes offer plausible explanations for the frequent reports of memory deficits, learning difficulties, and motor impairments among participants [20].

The mechanisms underlying alcohol‐related brain damage (ARBD) are diverse and interrelated, involving excitotoxicity due to excessive glutamate activity, oxidative stress, neuroinflammation, mitochondrial dysfunction, and apoptosis [21, 22, 23]. Furthermore, chronic alcohol use impairs thiamine absorption and metabolism, precipitating conditions such as Wernicke's encephalopathy and Korsakoff syndrome, which exacerbate cortical degeneration, particularly in malnourished individuals [20, 24]. These effects are amplified in low‐resource settings like Angola, where nutritional deficiencies, delayed access to healthcare, and the absence of neurorehabilitation services remain persistent challenges.

Psychiatric and neurobehavioral manifestations of ARBD were also evident in this study. The strong association between heavy drinking and symptoms such as aggression, tremors, and excessive sleepiness aligns with evidence that alcohol disrupts the hypothalamic–pituitary–adrenal (HPA) axis and impairs limbic regulation, leading to emotional dysregulation and impulsive behavior [25, 26]. These impairments contribute not only to personal dysfunction but also to broader familial and social breakdown.

Social stigma and unemployment were significantly associated with heavy alcohol consumption in our sample. Harmful alcohol use impairs occupational functioning, contributes to job instability, and reinforces social exclusion [27]. In Angola, as in many African countries, the intersection of youth unemployment and alcohol use represents both a consequence and a driver of marginalization. Cultural perceptions that frame alcohol dependence as a personal failure, rather than a neuropsychiatric disorder, may further entrench stigma and discourage treatment‐seeking behaviors [28, 29, 30].

Interestingly, the low reporting of family disruption contrasts with the severity of neurological and psychosocial symptoms observed. This may reflect underreporting due to social desirability bias or cultural norms that emphasize family cohesion, even when functional relationships are compromised. In collectivist societies, familial support often persists despite underlying emotional strain, and the lack of specialized mental health services may further obscure the true extent of alcohol‐related family dysfunction.

The findings reinforce the need for a multi‐pronged public health response. Evidence‐based interventions—such as increasing alcohol taxation, regulating marketing, enforcing age restrictions, and implementing school‐based prevention—have been effective in reducing harmful alcohol use in various contexts [31, 32]. However, in Angola, implementation is hampered by weak enforcement mechanisms, limited political prioritization, and under‐resourced health infrastructure.

Community‐level interventions that integrate health education, vocational training, and peer mentorship have shown promise in similar low‐resource settings, promoting resilience and reducing relapse rates [33, 34]. Additionally, neurocognitive rehabilitation programs targeting executive function, working memory, and behavioral control are essential components of recovery from alcohol‐related brain injury [35].

Finally, future research should adopt longitudinal designs to monitor the progression of alcohol‐related neurodegeneration and evaluate the effectiveness of culturally adapted harm‐reduction strategies. Particular attention should be given to sex‐specific pathways, given the marked gender disparity in alcohol use, and to the role of family systems in both risk and recovery. Collaborative partnerships between universities, the Ministry of Health, and civil society are crucial to designing and sustaining locally relevant interventions that address this growing public health challenge. The data supporting the findings of this study are available within the article and its supporting materials.

## 5 Conclusion

Conclusion: Excessive alcohol consumption among young adults in Luanda is strongly associated with male sex, lower educational attainment, and the consumption of high‐alcohol‐content beverages. Neuroimaging revealed significant parietal cortical atrophy among heavy drinkers, with corresponding neurological and psychosocial consequences including memory loss, tremors, aggression, stigma, and unemployment. Given the severity and complexity of the issue, there is an urgent need for integrated public health strategies that address both prevention and intervention. These should include school‐based education programs, community outreach, early screening for risky alcohol use, and the development of specialized services for neurocognitive rehabilitation. Implement school and community‐based alcohol prevention programs tailored to youth, enforce existing laws on alcohol sales and advertising, particularly targeting high alcohol content beverages, integrate early screening for risky alcohol use into primary healthcare, develop low‐cost neurocognitive rehabilitation services accessible to young adults, and launch stigma‐reduction campaigns to encourage treatment‐seeking. Future research should be longitudinal and community‐based to monitor neuroanatomical changes over time. Collaboration between government agencies, academic institutions, and civil society will be essential to reduce the burden of alcohol‐related harm and improve the health and prospects of Angola's youth.

## Author Contributions

**Bevaldo de Oliveira Guerra:** conceptualization, investigation, writing – original draft, methodology, visualization, formal analysis, data curation. **Celestino Delgado:** visualization, validation, supervision, resources, project administration, conceptualization. **Euclides Nenga Manuel Sacomboio:** conceptualization, investigation, funding acquisition, writing – original draft, methodology, validation, visualization, writing – review and editing, software, formal analysis, project administration, data curation, supervision, resources.

## Conflicts of Interest

The authors declare no conflicts of interest.

## Transparency Statement

The lead author Euclides Nenga Manuel Sacomboio affirms that this manuscript is an honest, accurate, and transparent account of the study being reported; that no important aspects of the study have been omitted; and that any discrepancies from the study as planned (and, if relevant, registered) have been explained.

## Supporting information

GLOSSÁRIO Supplementar Bevaldo.

## Data Availability

The data that support the findings of this study are available from the corresponding author upon reasonable request. The authors confirm that the data supporting the findings of this study are available within the article and its supporting information. Additional data may be made available upon reasonable request to the corresponding author.
