# Supplementary material for: Neuroanatomical and Psychosocial Effects of Heavy Alcohol Use Among Young Adults in Angola: A Cross‐Sectional Study
Source: Health Sci Rep. 2025 Oct 6;8(10):e71358. doi: 10.1002/hsr2.71358 (PMC12500527; doi:10.1002/hsr2.71358)
Supplement: Supplementary file 1 — GLOSSÁRIO Supplementar Bevaldo. [file HSR2-8-e71358-s001.docx]

**Supplementary Material**

**Neuroanatomical Changes and Psychosocial Effects of Heavy Alcohol Use Amog Young Adults in Angola: A Cross-Sectional Study**

**Running title:** Neuroanatomical Changes and Psychosocial Effects of Heavy Alcohol Use Among Young Adults

Author´s list

**Bevaldo de Oliveira Guerra^1^ Celestino Delgado^2^  Euclides Nenga Manuel Sacomboio^3^**

Author´s affiliations

^1^ Graduated from the Faculty of Medicine of the Jean Piaget University of Angola, Luanda-Angola. valdospark@gmail.com

^2^ Specialist in Imaging, professor at the Faculty of Medicine of the Jean Piaget University of Angola, Luanda-Angola. celestinoscp@gmail.com

^3^ Ph.D. Professor and researcher at the Institute of Health Sciences of the Agostinho Neto University (ISCISA/UAN), Luanda-Angola. General Director of the Higher Institute of Health Sciences of the Catholic University of Angola (ISCS/UCAN), Luanda-Angola. euclides.sacomboio@uan.ao;

*Address correspondence to Euclides Sacomboio, Instituto Superior de Ciências da Saúde, Universidade Católica de Angola. E-mail: [euclides.sacomboio@ucan.edu](mailto:euclides.sacomboio@ucan.edu)

ORCHID

Bevaldo de Oliveira Guerra: 0000-0003-4849-0267

Celestino Delgado: 0009-0001-8193-1071

Euclides Sacomboio: 0000-0002-2341-9133

**GLOSSARY**

Tobacco (Tumbaco) is a brown substance in the form of a powder similar to coffee. Its origin is dehydrated tobacco leaves, ground into a powder similar to coffee. The powder is inhaled and, according to users, causes a burning sensation in the airways, watery eyes, tinnitus, dizziness, headache, and decreased motivation when used for the first time. In Angola, the term Tumbaco derives from the Lingala language, commonly used by young Congolese in Angola and currently spoken by young people native to the country.

Liamba, from the English (Weed/Marijuana, cannabis), from the Brazilian (Maconha) in Angola is known as "Stufa Paciente," "Pica," or "Djamba." Its association with drugs such as crack is called "Speedbau."

Crake is called "Latinha" (Latin) due to its use, which is melted and smoked in a soda can apparatus.

Alcoholic beverages, both spirits and distilled, are often referred to as "pacotinhos" (little packets), a reference to the current form, which is packaged in 50mL packets.

The Angolan drink, formerly known as "Caporroto," is now known as "Água do Chefe" (Chief's Water).

The drink known as "Jeta" is another alcoholic beverage not approved for consumption, as it is a mixture of several distilled alcoholic beverages, with Múcua juice, and jet fuel. When mixed together, it is called "Caipirinha do Azar" (Caipirinha of Bad Luck), a name coined by young people due to the harmful effects it caused during the period of its commercialization and consumption.

**APPENDICES**

**Figure 1: CT, axial MPR, normal anatomical pattern.**
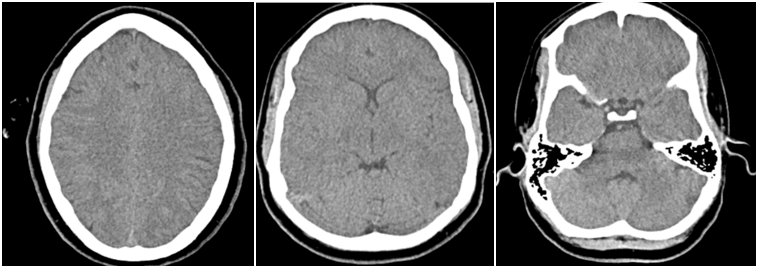
**Source: (Participants' CT Scans, 2023)**

A

A

A

**Figure 1 – Axial MPR Cranial CT Scan: Normal Neuroanatomical Pattern**

**Image A:**

This image illustrates the standard neuroanatomical pattern of 23-year-old male, healthy young adult without a history of heavy alcohol or psychoactive substance use. The cortical sulci and gyri are well defined and symmetric, and the cerebral hemispheres exhibit a normal and age-appropriate volume. No evidence of cortical atrophy, hypodense lesions, or structural deformities is observed. Subcortical structures and ventricular morphology appear preserved. The nasal cavity and turbinates also maintain their normal architecture and density, serving as an essential baseline for comparison with the pathological alterations presented in subsequent figures.

**Figure 2: CT of the skull, axial plane MPR, frontal atrophy, and changes in the middle turbinates.**
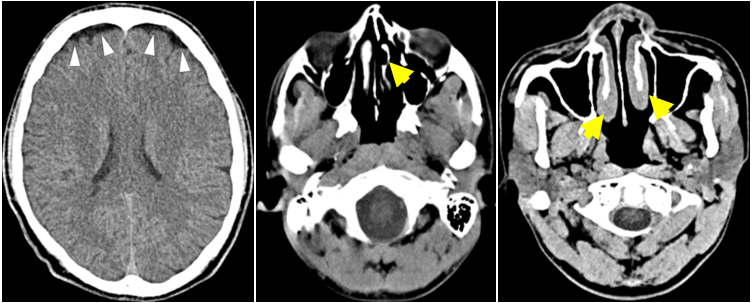


D

C

B

**Source: (Participants' CT Scans, 2023)**

**Figure 2 – Axial MPR Cranial CT: Frontal Lobe Atrophy and Middle Turbinate Alterations**

**Image B:**

Cranial CT scan of a 25-year-old male with a documented history of heavy and concomitant use of alcohol, tobacco, marijuana, cocaine, and snuff (tumbaco). Marked bilateral frontal lobe atrophy is evident (white arrows), characterized by enlargement of cortical sulci and reduction in frontal gray matter volume, indicative of significant neuronal loss. These structural changes are often associated with deficits in executive function, impaired judgment, impulsivity, and reduced cognitive flexibility — all functions primarily mediated by the frontal lobes.

**Image C:**

The same patient shows a circular hypodense lesion (yellow arrow) at the level of the middle nasal turbinates, consistent with tissue destruction or mucosal necrosis. Chronic inhalation of cocaine and tobacco may induce local vasoconstriction, ischemia, and persistent inflammatory damage, leading to progressive mucosal degeneration and potential compromise of nasal physiology.

**Image D:**

Cranial CT of a 22-year-old male with a history of heavy alcohol, tobacco, and marijuana use. There is clear evidence of diffuse thickening of the middle turbinate mucosa (yellow arrows), suggesting a chronic inflammatory response due to repeated chemical irritation. Such mucosal hypertrophy is associated with chronic rhinitis, recurrent infections, and nasal obstruction, which are frequent complications in chronic inhalant users.

**Figure 3: CT scan of the skull, axial MPR, temporal and cerebellar atrophy.**


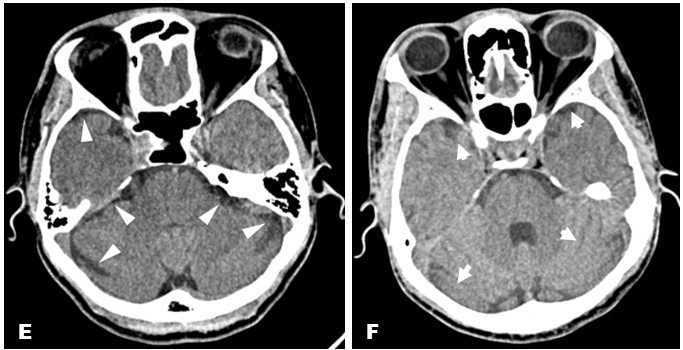


**Source: (Participant CT Scans, 2023)**

**Figure 3 – Axial MPR Cranial CT: Temporal and Cerebellar Atrophy**

**Image E:**

CT scan of a 32-year-old male, heavy smoker with no reported use of illicit substances. Marked atrophy of the left temporal lobe (white arrows) is visible, along with cerebellar volume loss and slight dilatation of the fourth ventricle. Temporal lobe degeneration is often linked to memory deficits, language disturbances, and impaired auditory processing, while cerebellar involvement can result in motor coordination impairments and balance disorders.

**Image F:**

CT scan of a 30-year-old male, chronic heavy alcohol consumer without other substance use. Bilateral temporal lobe atrophy is evident, accompanied by significant cerebellar degeneration and widening of cerebellar sulci — findings consistent with advanced neurodegeneration. Chronic alcohol exposure exerts cumulative neurotoxic effects, leading to neuronal loss, axonal degeneration, and functional decline in regions essential for cognitive processing, memory consolidation, and motor coordination.

**Figure 4: Skull CT, axial MPR, parietal atrophy, and destruction of the right turbinates.**


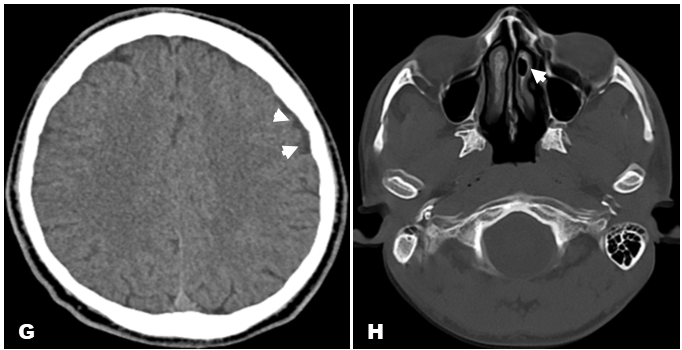


**Source: (Participants' CT Scans, 2023)**

**Figure 4 – Axial MPR Cranial CT: Parietal Atrophy and Right Turbinate Destruction**

**Image G:**

Cranial CT of a 25-year-old male with a history of heavy alcohol consumption combined with illicit drug use. Severe frontoparietal cortical atrophy is observed, evidenced by marked sulcal widening and significant cortical volume reduction. These areas are involved in sensory integration, attention regulation, and executive control. Such structural degeneration is often associated with slowed cognitive processing, attentional deficits, and impaired working memory.

**Image H:**

In the same patient, the scan shows extensive destruction of the right nasal turbinate membranes, indicating advanced mucosal damage likely secondary to chronic inhalation of toxic substances. Persistent vasoconstriction, ischemic injury, and inflammatory necrosis result in the loss of nasal architecture, compromising essential physiological functions such as air filtration, humidification, and temperature regulation, while significantly increasing the risk of recurrent respiratory infections.

**Summary of Figures 1–4**

These images collectively demonstrate a progressive spectrum of neuroanatomical and sinonasal alterations associated with chronic and heavy alcohol and substance use. Compared to the normal pattern (Figure 1), pathological findings include frontal, temporal, parietal, and cerebellar cortical atrophy, mucosal thickening, turbinate destruction, and hypodense lesions indicative of neuronal loss and tissue necrosis. The observed structural changes reflect complex neurotoxic processes, including neuronal degeneration, demyelination, chronic inflammation, and ischemic injury, which synergistically contribute to cognitive decline, behavioral alterations, and sensory-motor deficits in affected individuals.
